# 

**Published:** 1878-01

**Authors:** 


### CONTENTS OF VOL. XXXII.

**A.**

A New Dental Journal............ 40

A Peculiar Case of Irritation of the Nervous System in a Child while Teething......... 76

American Dental Convention.... 434

Annual Address. By A Wilkes Smith ............................ 323

Alumni Re-union.................... 307

Analysis of Normal Saliva...... 229

American Dental Association... 263

A New Dental College............ 263

Annual Address...................... 133

Address. By Geo. L. Field...... 177

" " Dr. Jas. Taylor... 184

" " H. E. Beach........ 192

Another Americen Dentist for Europe ............................ 211

A Law Regulating the Practice of Dentistry in Kentucky... 217

A New Theory of the Nature of Water ............................ 125

A Bill Relating to Dental Practitioners in Great Britain... 126

A New Medical Journal.......... 130

A New and Good Move........... 260

Arsenious Acid in Dentistry. By J. Hardman............... 499

An Index Rerum of Dental Literature. By D. C. Hauxhurst................................ 509

**B.**

Board of Dental Examiners...... 44

"Bleaching Teeth".................. 386

**C.**

Conservation of Vital Force. By Dr. I. Williams............ 33

Cyanide of Zinc in Facial Neuralgia........................ 41, 125

Cincinnati Bi-Ennial Musical Festival......................... 129

Commencement................ 171, 172

Correspondence.. 208, 348, 431, 432

Conn. Valley Dental Society..... 261

Cases of Infantile Paralysis...... 60

Celluloid.............................. 73

California State Dental Association ................................ 173

Close of the Volume............... 522

Correction............................. 524

**D.**

Dental Education.................... 45

Draft of a Bill to Amend the Law Relating to Dental Practitioners in Great Britain.................................. 107

Dangerous Colors in Wall Papers ................................ 125

Dental Law........................... 129

3375-37

INDEX.

Discussions Before the Miss. Valley Dental Association....... 152

"Do we use Diamond Disks?"... 213

Discussions before the Kentucky State Dental Society... 298, 330

Diagnosis. By L. C. Ingersoll... 353

Disease of the Gums................ 396

Diamond Disks........................ 433

E.

Eastern Indiana Dental Association................................ 175

Editorial, 40, 84, 126, 213, 257, 305 349, 386, 433

Erratum........................... 87, 352

Embryology. By Dr. W. H. Atkinson.......................... 367

F.

From the Viertejahresschrift..... 220
Filling Teeth.......................... 74

G.

Galvanic Action Within the Mouth. Geo. Watt............ 23

H.

Histology and Microscopy. W. H. Atkinson..................... 89

History of Dentistry................. 172

Ho, to American Dental Association................................ 310

Hard Rubber Appliance for Congenital Cleft Palate. By Thomas Brain Gunning...... 513

I.

Illinois State Dental Society..... 174

Iowa " " " 215

Interglobular Spaces. By D. C. Hauxhurst...................... 221

J.

Journalistic Consolidation........ 351

K.

Kentucky State Dental Society.. 213

L.

Local Anæsthesia.................... 433

List of Societies...................... 522

M.

Michigan Dental Association... 127

Mississippi Valley Dental Association......................... 84, 176

Material for Filling Teeth. By Dr. I. P. Wiison............... 397

Medical and Surgical Society of Baltimore........................ 65

Man's Average Height........... 79

Materials for Fillings. De-Ce-Aich.............................. 512

N.

Nebraska State Dental Society... 41

Nerve Stretching for the Relief of Neuralgia.................... 211

National Association.............. 257

O.

Opium.................................. 125

Oral Secretions. By G. S. Shattuck.............................. 225

Oral Electricity. By J. S. Cassidy.............................. 265

Obituary.................... 85, 86, 310

P.

Poisoning by Burning Gas........ 42

Practical Dentistry. By W. C. Duncan, D. D. S............... 149

Portland Cement a Material for Filling............................ 219

Practical Remarks on the Doses of Medicine...................... 240

Proceedings of the Ohio Dental College Association........... 254

Professional Duties of the Dentist. By C. L. Kelsey........ 278

Penn. State Dental Society........ 305

Proceedings of the 19th Annual Meeting of the Northern Ohio Dental Association 421 340

Proceedings of the Iowa State Dental Society.................. 410

Proceedings of the Wisconsin State Dental Society.......... 427

Pivot Teeth.......................... 271

Prevention of Disease, Self-Abnegation.......................... 59

R.

Retaining Points..................... 70

Resignation and Reorganization, 309

Report on Therapeutics. By J. T. McMillan.................... 311

Relief for the Dentist.............. 435

Removal of Strong Odors from the Hands.............. ........ 80

S.

Selections ................................ 125

Surgery of the Face. By Francis Mason...................... 233

Surgery.............................. 250

Southern Dental Association..... 261

Surgical Operations on the Pulp, and the Best Means of Extracting it. By Dr. C. G. Edwards.......................... 317

Something New About Oxygen.. 79

Something New..................... 523

T.

The Effects of Anæsthetics upon the Human System, as Evidenced by Spectroscopic Observations. By Dr. S. Waterman....................... 9

The Dental and Oral Science Magazine....................... 131

The Thirty-Fourth Annual Meeting of the Mississippi Valley Association of Dental Surgeons..................... 205

The Dentists' Chair................ 349

Treatment of Serous Effusions by Limitation of Fluid...... 252

The Power of Aerostatic Changes upon Abnormal Tissue. By E. S. Chisholm,.................. 483

The Dental Pulp. By Wm. Pease.............................. 487

Transplanting. By G. R. Thomas D. D. S.......................... 281

Therapeutical Treatment of Dental Pulps. By Dr. McLain 376

The American Dental Association.............................. 394

Transactions............................ 396

Therapeutical Treatment of Dental Pulps........................ 405

The Science of Temperence...... 81

The Ohio Dental College Association............................ 85

V.

Vick's Illustrated Monthly Magazine............................ 131
Virginia State Dental Society... 524

W.

Why is Hygiene not Taught in Dental Colleges?............... 42

Wisconsin State Dental Society, 259

### CONTENTS.

A MONTHLY MAGAZINE DEVOTED TO THE INTERESTS OF

**THE DENTAL PROFESSION.**

*TERMS REDUCED TO $2.50 PER YEAR. SINGLE COPIES 25C.*

EDITED BY PROF. J. TAFT, D.D.S.

AND

*Published by SPENCER, CROCKER & CO., Ohio Dental Depot.*

The volume commences in January and closes in December.

The REGISTER being issued monthly is always fresh, therefore Indispensable to the practitioner who desires to keep posted up with the new inventions and improvements which are daily developed. Neither expense nor effort will be spared to give value and variety to its contents. Articles will be illustrated with well executed engravings whenever they will add to the interest or assist to explanation.

Its extensive circulation and frequency of issue make it one of the BEST DENTAL ADVERTISING MEDIUMS in the United States.

All subscriptions must begin with January or July.

Local or special notices, five lines or less, will be inserted for$3 each insertion; over five lines, 50 cts. per line.

All advertising bills payable in advance, or at furthest, after the issue of the first number containing the advertisement. All transient adver-

